# Reconstruction of Large Osteochondral Lesions in the Knee: Focus on Fixation Techniques

**DOI:** 10.3390/life11060543

**Published:** 2021-06-10

**Authors:** Christian D. Weber, Filippo Migliorini, Frank Hildebrand

**Affiliations:** 1 Department of Trauma and Reconstructive Surgery, RWTH Aachen University, Pauwelsstr. 30, 52074 Aachen, Germany; fhildebrand@ukaachen.de; 2 Department of Orthopaedics and Trauma Surgery, RWTH Aachen University, 52074 Aachen, Germany; migliorini.md@gmail.com

**Keywords:** large osteochondral lesion, flake fracture, articular fracture, osteochondral defect, knee joint, reconstructive techniques

## Abstract

Large (>3 cm^2^), focal osteochondral lesions (OCL) may result in poor functional outcomes and early secondary osteoarthritis of the knee. The surgical management of these OCL remains challenging. The treatment strategy must be tailored to various aspects, including lesion-specific (e.g., size, location, chronicity), joint-specific (e.g., instability, limb alignment, meniscal status), and patient-specific factors (e.g., age, activity level, comorbidities). Simple chondroplasty and bone marrow stimulation (BMS) techniques should be reserved for smaller lesions, as they only realize midterm clinical benefits, related to inferior wear characteristics of the induced fibrocartilage (type I collagen). Therefore, much attention has been focused on surgical restoration with hyaline cartilage (type II collagen), based on chondrocyte transplantation and matrix-assisted autologous chondrocyte implantation (MACI). Limited graft availability, staged procedures (MACI), and high treatment costs are limitations of these techniques. However, acute traumatic OCL of the femoral condyles and patellofemoral joint may also be suitable for preservation by surgical fixation. Early detection of the fragment facilitates primary repair with internal fixation. The surgical repair of the articular surface may offer promising clinical and cost-effective benefits as a first-line therapy but remains under-investigated and potentially under-utilized. As a unique characteristic, the fixation technique allows the anatomic restoration of the hyaline articular surface with native cartilage and the repair of the subchondral bone. In this manuscript, we present a case series of large OCL around the knee that were preserved by surgical fixation. Furthermore, various implants and techniques reported for this procedure are reviewed.

## 1. Introduction

Osteochondral lesions (OCL) most frequently occur around the knee joint. Large OCL of the knee affect young and active individuals especially and may occur either as an isolated injury or in association with a ligamentous injury [1,2,3,4,5]. The prevalence of osteochondral lesions in common knee injuries, e.g., anterior cruciate ligament (ACL) tears and patella dislocations, is significant [6,7,8]. 

Large, displaced OCL with an intact cartilaginous surface are considered as an indication for surgical treatment. In these cases, where the salvage of the large fragment by internal fixation seems to be feasible, the preservation of the native cartilage is the theoretically ideal, single-stage, cost-effective, first-line technique for the high-loading components of the knee. In very large osteochondral fractures, internal fixation may even be the only rational therapeutic approach or an instrument to downsize the lesion. 

The major principles of intraarticular fractures are also valid for the treatment of large OCL. These include the reconstruction of articular congruity, achieving stable fixation, restoring joint stability and allowing early joint motion [9]. Unfortunately, a significant proportion of these injuries are not diagnosed in the acute setting. For this reason, the existing body of literature is focused on the management of osteochondral defects, based on repair tissue stimulation, transplantation or regeneration of cartilage [10,11,12,13]. In the past literature, no consensus regarding the optimal management of OCL around the knee with one specific technique has been established, but a failure rate of 17% was reported [14]. 

A recent study suggests that in large osteochondral fractures after patella dislocation, internal fixation improves mid- and long-term outcomes when compared to debridement [15]. Furthermore, salvage techniques have been successfully applied even in late-diagnosed large fragments [16,17] or in chronic lesions, when combined with autologous bone grafting [18].

In light of the current literature, the spectrum of indications for internal fixation techniques for fragment preservation is evolving. We present the case-based management of three patients with large osteochondral fractures of weight-bearing knee components and review the current literature on salvage fixation techniques for these complex knee injuries. 

## 2. Material and Methods

### 2.1. Study Selection

**Figure 1:**
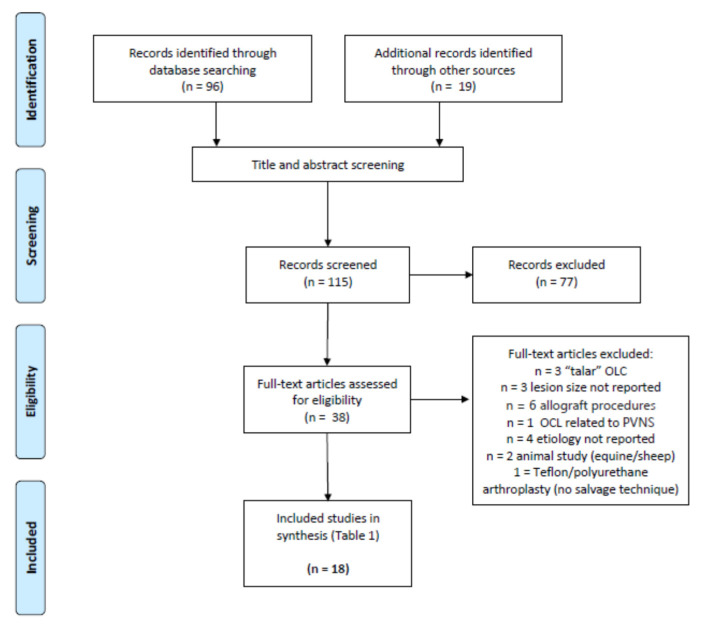
Study selection algorithm. Abbreviations: osteochondral lesion (OCL), pigmented villonodular synovitis (PVNS).

The study was conducted according to the guidelines of the Declaration of Helsinki, and approved by the local Ethics Committee (EK 224/21). Formal ethical statements of the included primary research may also be applicable. The study selection was not blinded for author, affiliation or source. An independent evaluation of the screened and discussion of the screened articles were performed by two reviewers (C.D.W., F.H.). The selection algorithm is presented in Figure 1.

### 2.2. Search Strategy

The literature acquisition was based on electronic databases only (PubMed/Medline, Embase, and the Cochrane Library). Suitable clinical trials, experimental studies, as well as review articles published between 1 January 1990 and March 2021 were considered for inclusion. Animal studies were excluded. The following terms were used in the search strategy:

1. Anatomic region affected: “knee” OR “patella” OR “femoral condyle” OR “trochlea” NOT “elbow”, OR “tibia”, NOT “ankle”, NOT “talus”
2. Entity/lesion type: AND “osteochondral lesion” OR “trans-chondral fracture” OR “osteo-/chondral” AND “lesion” OR “defect” OR “fracture” OR “flake” OR “injury” OR “fragment”
3. Surgical technique: AND “internal fixation” OR “repair” OR “fragment preservation” OR“ salvage” OR “reconstruction”
4. Lesion size (optional, if characterized): “large” OR “massive”

Articles published in the English or German language were considered for selection. Additionally, a backward citation chaining strategy was applied. 

## 3. Case Presentation

**Figure 2:**
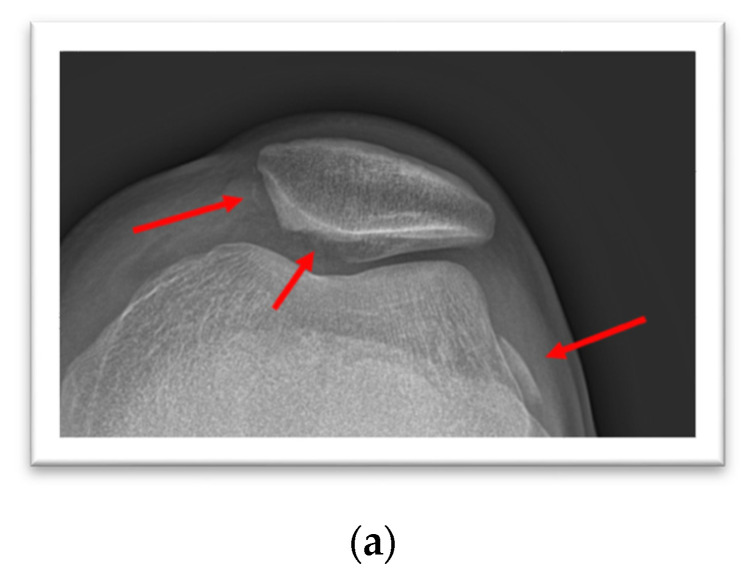

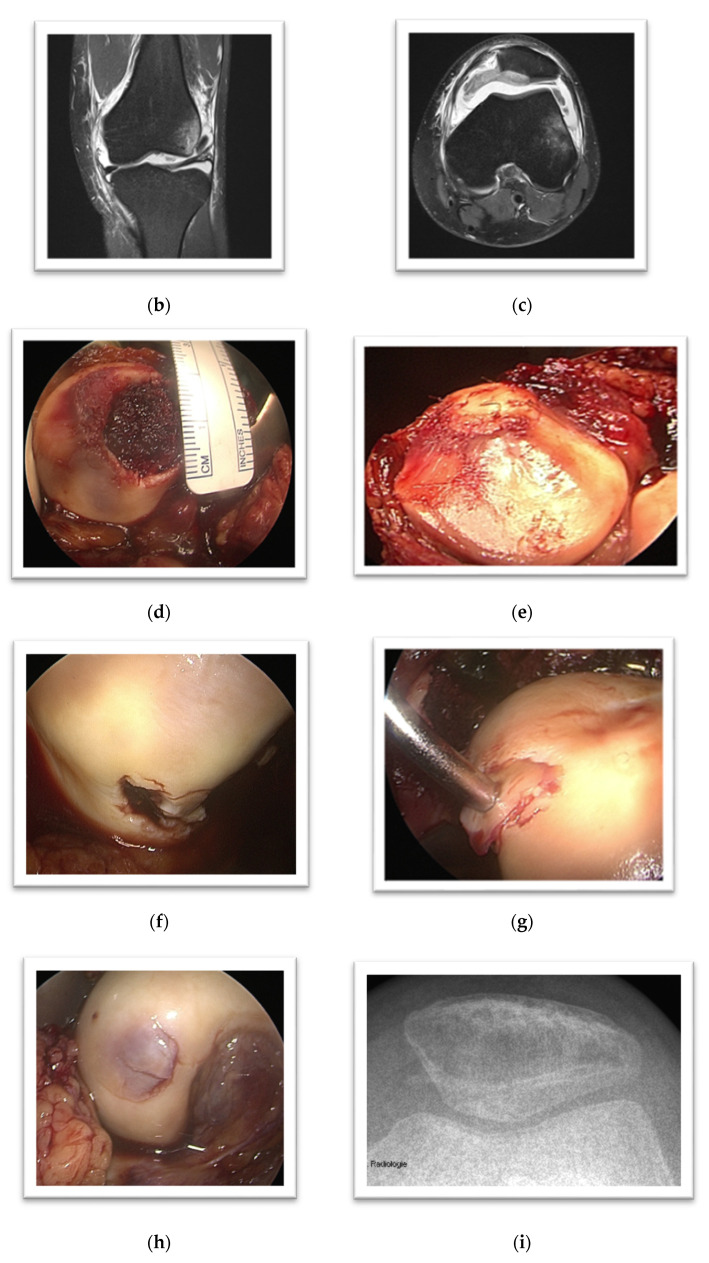

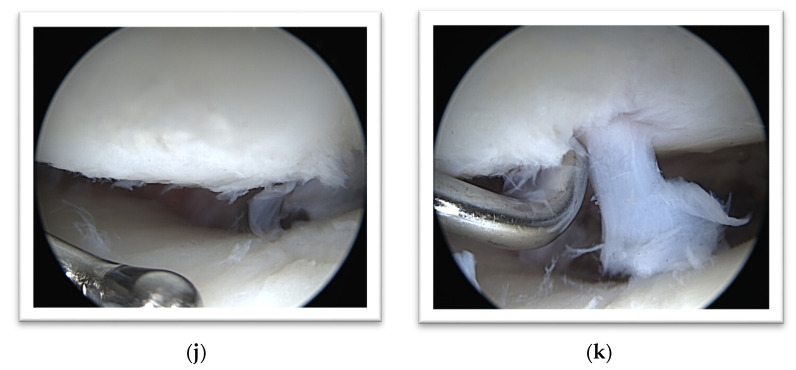
(**a**–**i**). Patella Dislocation. (**a**) Patella sunrise view, suggestive of MPFL and MPTL avulsion, a large osteochondral defect of the patella, and a displaced fragment in the lateral gutter. (**b**,**c**) MRI of the left knee, joint effusion, osteochondral fracture of the patella, displaced fragment in the lateral gutter, bone bruise of the lateral femoral condyle. (**d**–**h**) Intraoperative situation, osteochondral defect of the medial facet of the patella, before and after fragment fixation, small full-thickness osteochondral lesion of the lateral femoral condyle repaired with one chondral dart, partial-thickness chondral injury of the medial femoral condyle. (**i**) Postoperative patella sunrise view. (**j**,**k**). Arthroscopic re-evaluation three months postoperative: (**j**) the osteochondral lesion is healed and stable under probing, (**k**) the small remaining defect is filled with fibrocartilaginous tissue.

A 22-year-old male sustained a first-time patella dislocation in the left knee, and a large displaced fragment from the articular surface of the patella was identified radiographically (Figure 2a). A combined injury of the medial patellofemoral ligament (MPFL), medial patellotibial ligament (MPTL) and focal osteochondral lesions of the patellofemoral joint were confirmed in a subsequent magnetic resonance imaging (MRI; Figure 2b,c), and the large osteochondral fragment was located in the lateral joint space. The patellar fragment (2.0 × 2.0 surface area, Figure 2d) was fixed after arthrotomy with three chondral darts (Figure 2e, Chondral Flap Repair System, Arthrex Inc., Naples, FL, USA) and additional monocryl sutures (Ethicon Inc., Somerville, NJ, USA). The MPFL was reconstructed with a quadriceps tendon graft technique (not shown). A small OCL of the lateral femoral condyle (LFC) was internally fixed with one chondral dart (Figure 2f,g). A partial-thickness chondral injury of the medial femoral condyle (MFC) was treated conservatively (Figure 2h). Radiographs confirmed the anatomic reconstruction of the patellar articular surface (Figure 2i). After three months, a second-look arthroscopy confirmed the fragment stability with a small remaining defect (Figure 2j,k) covered with fibrocartilaginous tissue, while performing a revision for arthroscopic adhesiolysis. 

## 4. Case Presentation 

**Figure 3:**
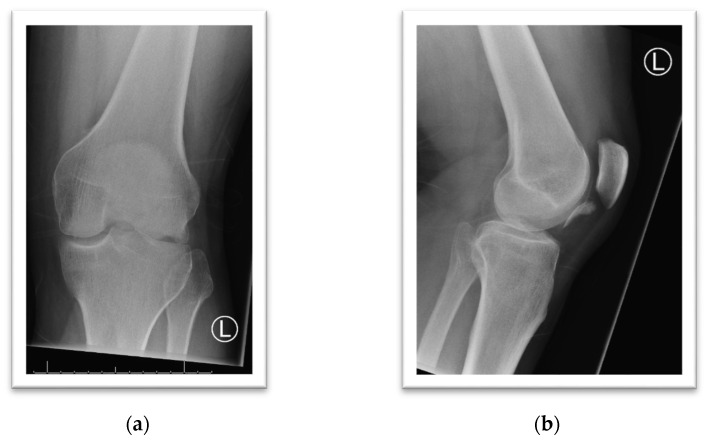

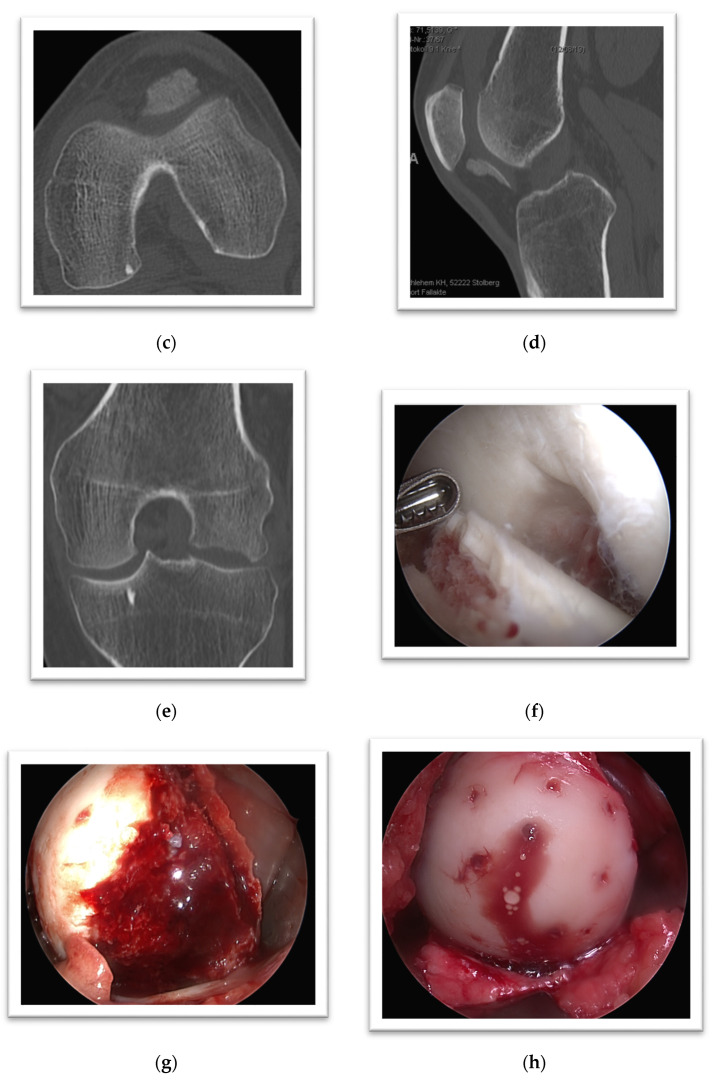

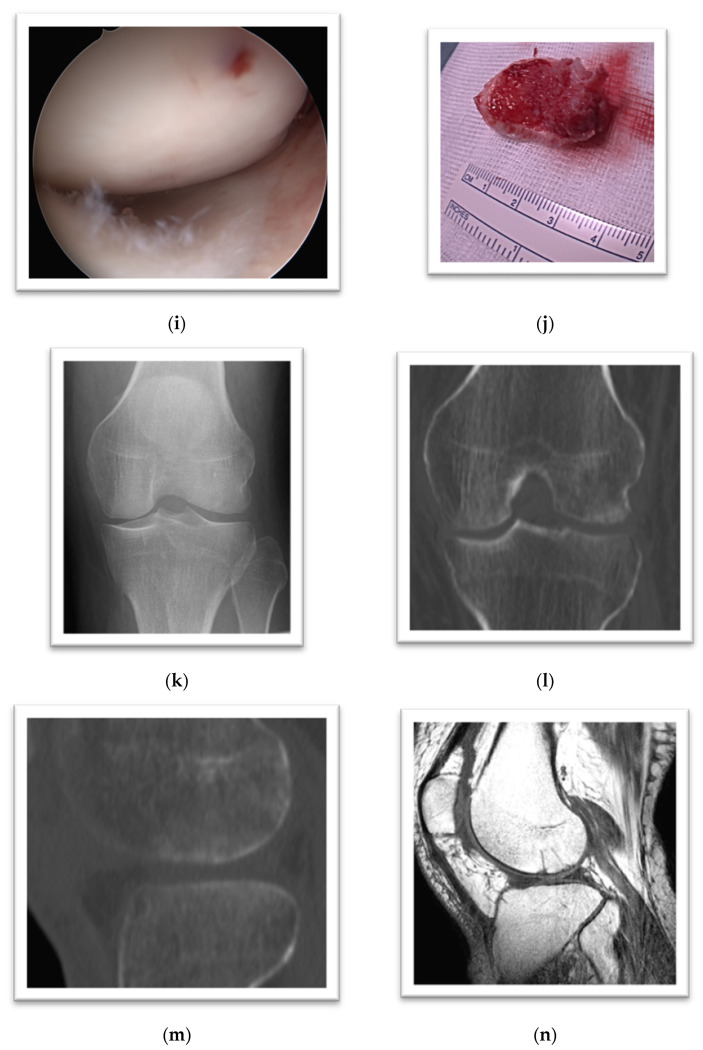
(**a**) Anterior-posterior radiograph of the left knee, limited range of motion due to the injury is obvious. (**b**) Lateral radiograph of the left knee, the osteochondral fragment is located inferior to the lower pole of the patella. (**c**–**e**) The coronal, axial and sagittal reconstruction during computed tomography (CT) reveals a massive osteochondral fracture of the lateral femoral condyle (LFC). (**f**) Arthroscopic evaluation of the displaced fragment. (**g**) The majority of the weight-bearing portion of the lateral femoral condyle is involved. (**h**) Reconstruction of the LFC after fixation with multiple PLLA darts. (**i**) Arthroscopic evaluation after internal fixation. (**j**) Osteochondral fragment with surface area measuring 3 × 2.8 cm, debridement was performed ex situ. (**k**) Postoperative AP radiograph shows anatomic articular alignment. (**l**,**m**) Sagittal and coronal CT reconstruction after three months shows adequate healing, but some subchondral sclerosis is present. (**n**) Sagittal MRI (T1) follow-up at six months, intact and vital cartilage surface, remaining small subchondral lesions at the sites of dart insertion.

A 49-year-old male sustained a first-time patella dislocation while walking. A large osteochondral defect involving the entire weight-bearing aspect of the lateral condyle is visible, and the fragment is locking the patellofemoral joint (Figure 3a–e). Arthroscopic-assisted internal fixation with absorbable darts was performed (Figure 3f–j; Chondral Flap Repair System, Arthrex Inc., Naples, FL, USA). The radiological follow-up shows the anatomic fragment position and healing of the subchondral bone three months post-injury, and the MRI after six months is presented (Figure 3k–n). 

## 5. Case Presentation

**Figure 4:**
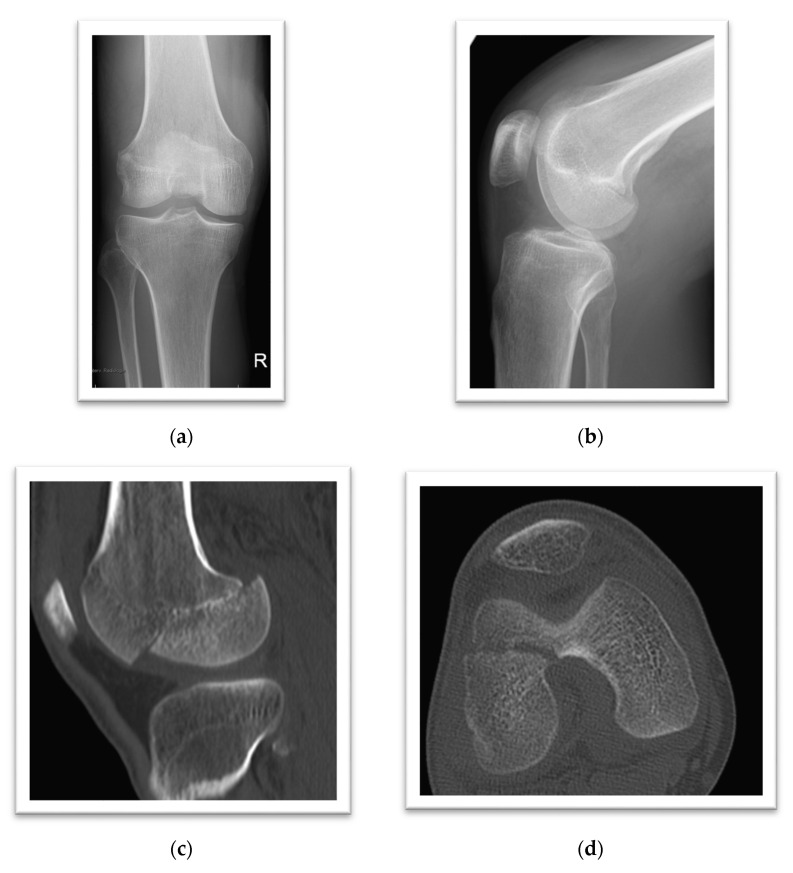

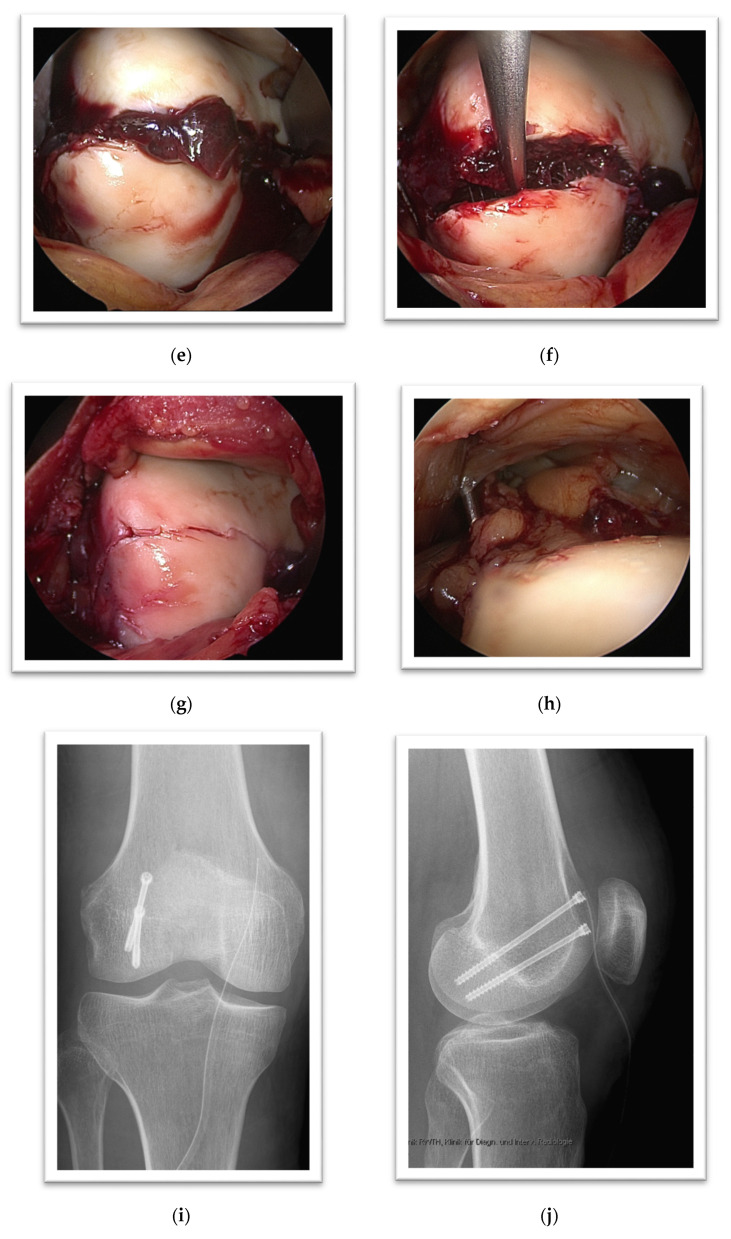

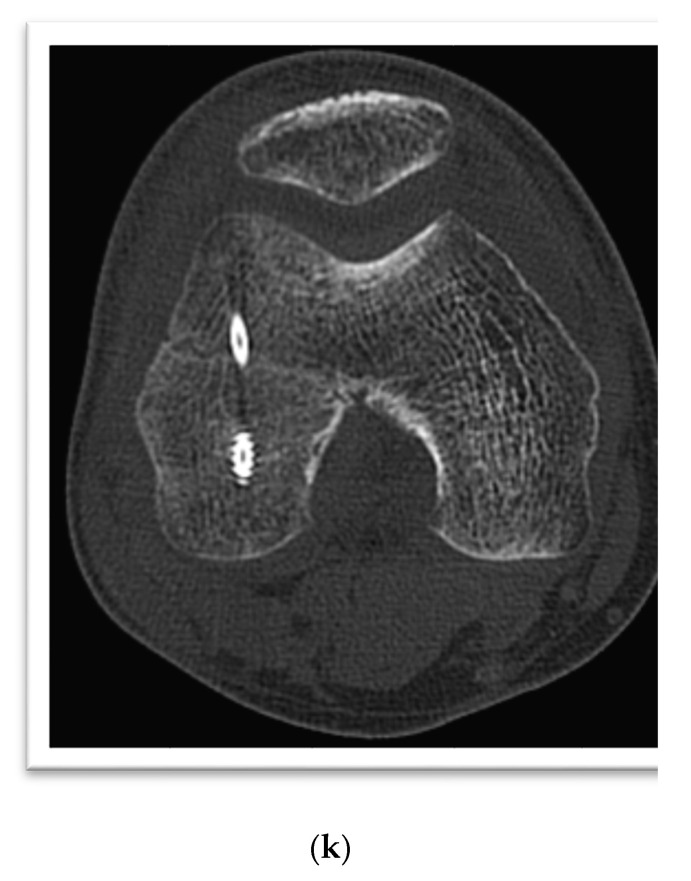
(a-k): (**a**) AP and (**b**) lateral radiograph of a Hoffa type fracture of the lateral femoral condyle, (**c**) sagittal and (**d**) axial assessment of the fracture morphology based on computed tomography (CT), (**e**) evaluation and (**f**) reduction for (**g**) anatomic reconstruction enables (**h**) arthroscopic assisted internal fixation; (**i**) AP and (**j**) lateral radiographic validation of screw positions and (**k**) follow-up CT.

A 34-year-old male sustained a high-energy motorcycle accident while the right knee was in a flexed position (Figure 4a,b). Axial and sagittal CT scans confirm the displaced injury with an articular step-off (Figure 4c,d). The majority of the lateral femoral condyle is fractured, attached only to the posterior capsule and partially to the ACL (Hoffa type, AO-33-B3). The displaced fracture (Figure 4e,f) was anatomically reduced and fixed internally in a percutaneous anterior-posterior fashion (Figure 4g,h) under arthroscopic assistance (4.0 mm partially threaded, Fixos^®^ headless compression screws, Stryker Inc., Kalamazoo, MI, USA). Proper visualization and identification of the ideal screw entry points preserving the femoral articular cartilage is facilitated under arthroscopic and fluoroscopic guidance (Figure 4h). The radiographic follow-up shows the anatomic position of the displaced fracture (Figure 4i,j) and adequate healing after three months via computed tomography (Figure 4k). 

## 6. Discussion

Both direct and indirect trauma mechanisms may result in osteochondral lesions around the knee [5,10]. Anterior cruciate ligament (ACL) tears are associated with injuries to the lateral femoral condyle, and osteochondral lesions of the patellofemoral joint are common following patella dislocations [19,20]. In this context, a recent study evaluated the patterns of osteochondral fractures after acute or recurrent patella dislocation and reported that 63% affected the patella, 34% affected the lateral femoral condyle and only 3% affected both locations [21]. This distribution can be explained by anatomic characteristics, as the articular cartilage of the patella is softer when compared to the trochlea, is thicker when compared to any other joint in the human body, and does not follow the contour of the subchondral plate. Despite multiple anatomic variations of the patellofemoral joint, these aspects increase the likelihood of osteochondral shear injuries under high biomechanical loading. Osteochondral fractures of the patella may be larger after primary dislocation than after recurrent dislocation, most likely due to increased contact pressures. Accordingly, in the two cases presented with a patellofemoral pathology, both patients experienced a first-time dislocation of the patella and sustained a large osteochondral fracture of the patella and the lateral femoral condyle, respectively. 

Song et al. evaluated an initial conservative treatment in sixty-nine patients with acute first-time patella dislocations. In the presence of large osteochondral fragments, the authors frequently observed a failure of the nonoperative treatment [22]. 

In the most recent treatment algorithms for patellar instability, recurrent patellofemoral instability or an osteochondral fracture are considered as an indication for surgical intervention [23,24]. In this context, Niemeyer et al. suggested that any osteochondral flake fracture indicates surgical treatment with the objective for internal fixation in both pediatric and adult patients [24]. Hence, due to the improved understanding of patellofemoral pathologies, a variety of arthroscopic and open surgical concepts for the repair of osteochondral lesions and the restoration of joint stability have been developed [25,26,27,28,29]. Furthermore, additional techniques for deformity correction (e.g., HTO) are inconsistently applied within the body of literature [30]. 

For patellofemoral pathologies, various chondral and osteochondral fixation techniques are suggested in combination with a wide spectrum of additional techniques, e.g., MPFL repair, MPFL reconstruction, medial reefing, lateral release. These methodological variances impede the scientific comparison of the reported study results. The reviewed literature predominantly comprises low level and quality evidence and is based on variable treatment protocols (Table 1). 

Furthermore, various methods of internal fixation are employed, including metallic (headless) and resorbable compression screws [23,24,26], Kirschner wires [26], resorbable polylactid implants (nails, pins and darts) [15,19,20,21,22,25,27,28,31,33], sutures (e.g., PDS) and suture-anchor constructs [29,34,35,47,48], fibrin sealants [19], and bone pegs [32]. Different main outcome measures and follow-up time periods are reported. To date, no consensus regarding a superior fixation technique or ideal characteristic for fragment preservation have been established. 

Meanwhile, the expertise for smaller OCL, e.g., around the talus, is expanding. For the ankle, a consensus opinion has been achieved for several therapeutic aspects, including the fact that surgical fixation can be considered for acute and chronic lesions with intact fragments larger than 10 mm and 3 mm thick, but this is contraindicated in cases of generalized osteoarthritis [49]. In terms of the timing of the surgery, the fragment should be fixed as soon as possible to maximize the healing potential and to reduce the risk of secondary articular damage. These strategic considerations might theoretically apply for both the ankle and the knee joint. 

As an example of a massive osteochondral fracture, the case of a Hoffa fracture has been illustrated (Case 3). This rare injury pattern consists of a very large osteochondral fracture of the femoral condyle in the coronal-plane and is most often related to high-energy trauma [50]. In general, vertical shear forces with varying degrees of knee flexion are responsible for this entity, which mandates surgical fixation [51]. Paradoxically, these large osteochondral fractures involving major portions of the femoral condyle (e.g., Hoffa fracture) may represent a diagnostic challenge because they may only be detected by the careful evaluation of the radiographs. In cases with inconclusive conventional radiographs, advanced imaging must be requested. Traditional surgical approaches are technically demanding and may involve a posterior buttress plate. The anatomic reduction of the articular surface and stable internal fixation are the principles of the surgical treatment. The arthroscopic-assisted reduction and minimally invasive internal fixation may improve outcomes, as an optimal screw placement can be validated and concomitant intraarticular injuries are identified arthroscopically [52,53]. 

These principles may also apply for large osteochondral lesions of the tibial plateau. However, the available literature on tibial lesions is sparse, and both osteochondral avulsion and impression fractures may occur at both the posterolateral (e.g., “apple-bite fracture”) and posteromedial tibial plateaus [54,55]. Melugin et al. recently performed a review focusing on cartilage lesions of the tibial plateau [56]. The reported surgical techniques included osteochondral allograft and autograft transplantation, microfracture, osteochondral scaffolds and autologous chondrocyte transplantation, but no salvage procedures based on fixation techniques. The authors observed heterogeneous patient-reported outcomes and even deteriorated midterm outcomes after microfracture. In light of these results, the preservation of available osteochondral fragments requires further evaluation. 

Major concerns in terms of fragment preservation remain in cases with chondral-only flaps and for those fragments which are only partially salvageable. Various authors reported a successful fixation and good outcomes even after the repair of chondral-only fragments, primarily in children and adolescents [22,25,31,32,35]. While some authors report that the subsequent swelling of the fragment is a concern in shear-off lesions because the anatomic reduction is impaired, a recent innovation takes advantage of the increased fragment dimensions. Jeuken et al. reported a modified Hedgehog technique in order to repair pure chondral shear-off lesions in pediatric knees [30]. Therefore, the chondral fragments were multiply incised and trimmed obliquely for an interlocking fit in the defect site, and the autograft was attached with fibrin glue and, if indicated, with sutures. However, the separation from the osteochondral unit is generally considered to be associated with a poor healing potential, especially in subacute cases and older patients. 

A new concept for partial preservation has been suggested for partially salvageable fragments by the Mayo Clinic and Osaka University, Japan [57]. The group recently described a “hybrid technique” for the fixation of a partial fragment with absorbable compression screws or PLLA pins and an osteochondral autograft transplantation system (OATS) for the remaining defect. The authors reported positive outcomes and no complications in patients with a mean lesion size of 2.8 cm2 after a follow-up of 36 months. 

In cases with a significant depression or fragmentation of large articular fragments, preservation may not be feasible at all [13]. For these cases, various techniques have been proposed, including (atelocollagen-associated) autologous chondrocyte implantation [58], the Mega-OATS technique [59], a combination of the osteochondral autograft transfer and the second-generation autologous chondrocyte implantation [60], as well as the minced cartilage implantation (MCI) procedure [61]. 

## 7. Conclusions

A high index of suspicion after acute knee injuries is vital for the early detection of large OCL, allowing the primary fixation of suitable fragments as a first-line therapy. Recent innovations including partial fragment preservation and “hybrid techniques” may increase the volume of salvaged hyaline cartilage and native subchondral bone. The wider application of arthroscopic techniques may be beneficial for the detection of concomitant pathologies, validation of anatomic fixation and improved outcomes. Compression and rotational fragment stability can be achieved by various surgical implants, and for some absorbable implants long-term outcomes are even available. For failed fixations, the management principles for osteochondral defects apply as second-line procedures. For specific treatment algorithms, a higher level and quality of evidence from future investigations is necessary. 

## Figures and Tables

**Table 1:** Summary of study results after internal fixation of larger osteochondral and chondral lesions around the knee.

Authors	Year	N	Age (y)	Fragment Composition	Lesion Size/Surface Area	Lesion Chronicity	Lesion Location	Fixation Device/Technique	Additional Procedures	Complications/Reoperations	Primary Outcomes Evaluated at Follow-Up
Maletius and Lundberg [31]	1994	2	18, 21	Osteochondral Chondral	2 × 3 cm 2.5 × 3.5 cm	Acute Chronic	Patella LFC	Fibrin sealant and polydioxanone pins	1 lateral release	2nd look arthroscopy + removal of detached fragm.	7/8 mo: symptom free, successful RTS
Dines et al. [32]	2008	9	18	Osteochondral	3.9 cm^2^	1 Acute 8 OCD	Femoral condyle	self-reinforced poly-L-lactic acid device (PLLA) nails		minimal complication rate	Lysholm score 94, excellent 7, good 1, fair 1
Walsh et al. [33]	2008	8	13.1	Osteochondral	>4.0 cm^2^	Acute	LFC (8)	multiple polyglycolic acid rods		4 s look arthroscopy, 1 unstable flap removal (20% of surface area)	Mean f/u 9 yr: Cincinnati Knee rating: 3 excellent, 2 good, 3 fair, no poor results
Chan et al. [34]	2014	1	12	Chondral	4 × 2.2 cm	Acute	LFC	1.5-mm-diameter polylactide fixation nails	MPFL repair	11.5 mo staged MPFL-R, healthy cartilage observed	Uneventful healing
Barrett et al. [35]	2016	22	21	Osteochondral	3.8 cm^2^	OCD	MFC (16) LFC (6)	Metal headless, cannulated compression screws		4 loose fragment and hardware removals: 1 wire breakage, 2 screw migrations	8.7 yr f/u: 82% union rate, IKDC 85, KOOS pain 93, KOOS ADL 98, KOOS Sports 82
Gesslein et al. [15]	2019	28	18.6	Osteochondral	3.7 cm^2^	Acute	Pat. (19) LFC (9)	1.3 mm BA pins	23 medial reefing	2 reinterventions	7.9 yr F/U, Lysholm 88.0, IKDC 89.2, KOOS Symptoms 87.2
Nuelle et al. [36]	2019	1	NR	Osteochondral	2.8 × 2.5 cm	Acute	Patella	3 mm PLLA compression screws		NR	Technical report only, outcome NR
Churchill et al. [37]	2019	10	14.6	Chondral	3.86 cm^2^	Acute	Trochl.(4) Patella (4) LFC (2)	2.7 mm BA or 2.4 mm metallic compression screws, PLLA nails or darts	2 MPFL repair	3 short-term complications: 2 screw migrations, 1 BMS for failure after 1.34 y	56 mo f/u: IKDC 94.74, Marx 14.4, TAS 7, 90% RTS
Wang et al. [38]	2019	45	14.9–18.3	Osteochondral	2.7–3.8 cm^2^	28 OCD	MFC (11)	BA and metal screws and/or Kirschner wires	ACLR 1, OTA 2, MMx 1	13 reoperations, 2 hardware migrations, 1 late BMS, 8 loose body removals	Kaplan–Meier survivorship 1-year 88.6% and 5-year: 68.8%
Schlechter et al. [39]	2019	38	14.7	Osteochondral	3.22 cm^2^	17 Acute 15 OCD	Femur (23) Patella (9)	3.0 mm PLLA compression screws and/or 1.3 mm darts	Staged ACLR/MPF 2 MUA	6 secondary proc., 1 for proud implant, none required cartilage revision proc.	59 mo: Lysholm 89.8, Pedi IKDC 88.1, Tegner postop. 6.4
Megremis [40]	2019	1	14	Osteochondral	>4 cm^2^	Chronic	Trochlea	1.5 mm BA pins			6 mo: Healing, successful RTS
Malecki et al. [41]	2019	17	14.1	Osteochondral	>1 cm^2^	Acute	Patella	Transpatellar PDS suture fixation	MPFL rep. if necessary	Degenerative changes in 3 patients	Mean f/u 7.5 yr: Lysholm 89.2, Kujala 89.6
Jeuken et al. [42]	2019	3	12.8	Chondral	2, 5, 8 cm^2^	Acute 1 Chronic 2	MFC (3), Trochlea (1)	Modified Hedgehog technique, fibrin glue, suture	1 ACLR	None at 1 yr f/u	12 mo: successful RTS, full ROM, no pain, stable fragment (MRI)
Beckert et al. [43]	2020	1	11	Chondral	2.2 × 2 cm	Acute	LFC	3 absorbable nails		Healing confirmed during arthroscopy	RTS after 6 mo, elite level at 7 mo
Ogura et al. [44]	2020	6	12.9	Chondral	3.8 cm^2^	5 Acute 1 OCD	Trochlea LFC	2.4 mm autologous bone pegs		1 failure 1.3 yr after a new injury	5.2 yr F/U, 85% success rate, RTS after 7 mo, excellent KOOS in 4
Rüther et al. [45]	2020	10	26.7	Osteochondral	3.33 cm^2^	10 Acute	Femur (2) Pat. (8) Tibia (1)	resorbable polylactid implants (screws, nails, pins)	7 med. repair, lat. release	NR	13.9 yr F/U: VASS 1.2, Lysholm 85.7, McDermott 90.7, Tegner 4.4
Zhou et al. [46]	2020	3	18	Osteochondral	1.5 × 1.5 cm	Acute	LFC	Titanium suture anchor and polydioxanone suture (PDS)		2nd-look arthroscopy in all patients	1 yr F/U: return to physical activity and uneventful healing
Gudemann et al. [30]	2021	15	17.7	Chondral	3.48 cm^2^	3 Acute 12 OCD	MFC (8) LCF (4) Pat. (3)	Screws (3), PDS suture (9), combination (3)	1 HTO	2nd-look arthroscopy showed loss of fixation in 3 knees	80% survival rate at 4 yr F/U, complete union in 67% (MRI)

Abbreviations: anterior cruciate ligament reconstruction (ACLR), Bone marrow stimulation (BMS), centimeter (cm), follow-up (F/U); high tibial osteotomy (HTO), International Knee Documentation Committee (IKDC), Knee Injury and Osteoarthritis Outcome Score (KOOS), lateral femoral condyle (LFC), Marx Activity Rating Scale (Marx), manipulation under anesthesia (MUA), medial femoral condyle (MFC), medial meniscectomy (MMx), medial patellofemoral ligament (MPFL), month (mo), millimeter (mm), not reported (NR), osteochondrosis dissecans (OCD), polydioxanone sutures (PDS), poly-L-lactic acid (PLLA), range of motion (ROM), return to sports (RTS), year (yr).
